# Supplementary material for: Combining serology with case-detection, to allow the easing of restrictions against SARS-CoV-2: a modelling-based study in India
Source: Sci Rep. 2021 Jan 19;11:1835. doi: 10.1038/s41598-021-81405-2 (PMC7815778; doi:10.1038/s41598-021-81405-2)
Supplement: Supplementary file 1 — Supplementary Information. [file 41598_2021_81405_MOESM1_ESM.docx]

Combining serology with case-detection, to allow the easing of restrictions against SARS-CoV-2: a modelling-based study in India

Sandip Mandal^1^, Hemanshu Das^2^, Sarang Deo^2^, Nimalan Arinaminpathy^3^*

^1^ Independent Consultant, New Delhi, India; ^2^ Indian School of Business, Hyderabad, India; ^3^ MRC Centre for Global Infectious Disease Analysis, School of Public Health, Imperial College London, London, UK

* Corresponding author: [nim.pathy@imperial.ac.uk](mailto:nim.pathy@imperial.ac.uk)

**Supporting technical information**

**Appendix 1: Model specification**

The model is a compartmental, deterministic framework, stratified by three age groups: <15yo, 15 – 64yo, and >65 yo. Figure S1 below illustrates the overall model structure, for a single age group.


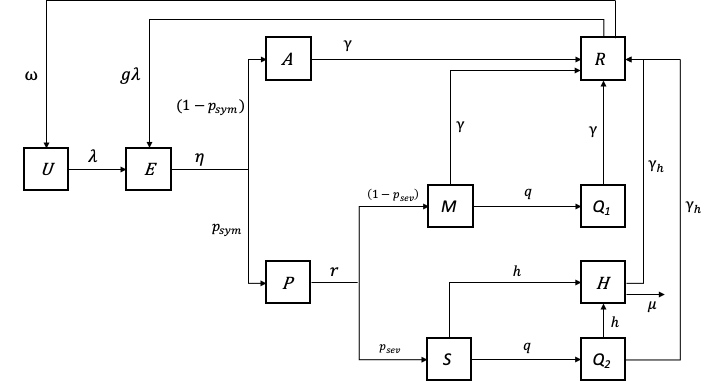


**Figure S1. Schematic illustration of the model structure**. State variable definitions (letters in boxes) are given in table S1, while parameter definitions (symbols on arrows) are listed in table S2. Governing equations relating to this diagram are listed in the text below.

In brief, the model incorporates major features of the natural history of SARS-CoV-2 infection: a period prior to developing symptoms, during which individuals are infectious (P); a proportion of cases being asymptomatic yet infectious (A); and a certain proportion of symptomatic cases (M, S) developing sufficiently severe disease that they require hospitalisation (H). The structure shown in Figure S1 was stratified by different age groups: <15 years old (yo), 16 – 64yo, and >65yo. To capture mixing between age groups, we drew from age-specific contact matrices that have been estimated for India (see terms $c_{ij}$ in Table S2). Amongst other features, the model also allows for waning of infection-induced immunity (as represented by the transition arrow labelled $\omega$ from recovered (R) to susceptible (S) compartments), as well as the potential for reinfection even amongst those with infection-induced immunity (as labelled by the transition arrow labelled $g\lambda$).

For illustrative results shown in Figs.1 – 2 in the main text, we adopted the assumed parameter values shown in Table S2. However, there remains much uncertainty about parameters relating to SARS-CoV-2 natural history, for example for the proportion of infections that are symptomatic: accordingly, for the main results of this study reported in Figs. 3 – 4, we adopted a wide range of parameter values to reflect this uncertainty (see Table 1, main text). The distribution of simulation outcomes shown in Figs. 3 - 4, main text, reflect this uncertainty, being propagated from model inputs to model projections.

An epidemic was simulated by seeding 10 infectives in a fully naïve population. The governing equations listed below were then simulated forward in time, initially in the absence of a lockdown. There is insufficient community-level surveillance data to inform the timing of the lockdown, relative to the epidemic (see Table 1). We adopted a range of scenarios, shown in Table 1 in the main text. We assumed that the effect of the lockdown is to diminish the overall rate of contact in the population, amounting to a reduced rate-of-infection $\beta^{'}=\beta(1-c)$, where *c* is the effectiveness of the lockdown in reducing transmission (see Table 1). We assumed a linear transition from $\beta$ to $\beta^{'}$ over a duration of 10 days starting from the time-of-initiation of the lockdown. Similarly upon lifting of the lockdown, we assumed a linear transition from $\beta'$to $\beta$, over a duration of 10 days starting from the time-of-lifting of the lockdown.

*Governing equations*

In the following equations, subscript *i* denotes the age group, with *i* = 1, 2, 3 denoting respectively, the age groups <15yo, 16 – 64yo, and >65yo.

Uninfected (*U*)

$$\frac{dU_{i}}{dt}=-\lambda_{i}U_{i}+\omega R_{i}$$

Exposed to infection (*E*)

$$\frac{dE_{i}}{dt}=\lambda_{i}U_{i}+g\lambda_{i}R_{i}-\eta E_{i}$$

Asymptomatic and infectious (*A*)

$$\frac{dA_{i}}{dt}=\eta(1-p^{(sym)})E_{i}-\gamma A_{i}$$

Presymptomatic and infectious (*P*)

$$\frac{dP_{i}}{dt}=\eta p^{(sym)} E_{i}-r P_{i}$$

Mild symptomatic (*M*)

$$\frac{dM_{i}}{dt}=r \left( 1-p_{i}^{(sev)} \right)P_{i}-(\gamma+q) M_{i}$$

Severe symptomatic (*S*)

$$\frac{dS_{i}}{dt}=r p_{i}^{(sev)}P_{i}-(h+q) S_{i,2}$$

Under quarantine (*Q*). Secondary subscripts denote severity, distinguishing mild symptomatic (1) from severe (2).

$$\frac{dQ_{i,1}}{dt}=q M_{i}-\gamma Q_{i,1}$$

$$\frac{dQ_{i,2}}{dt}=q S_{i}-\left( h+\gamma_{i}^{h} \right)Q_{i,2}$$

Needing hospitalisation (*H*)

$$\frac{dH_{i}}{dt}=h S_{i}+h Q_{i,2}-(\mu_{i}+\gamma_{i}^{h})H_{i}$$

Recovered and immune (*R*)

$$\frac{dR_{i}}{dt}=\gamma A_{i}+\gamma M_{i}+\gamma Q_{i,1}+\gamma_{i}^{h} H_{i}+\gamma_{i}^{h} Q_{i,2}-\left( \omega+g \right)R_{i}$$

Finally for the force-of-infection $\lambda_{i}$ in age group *i*, we have:

$$\lambda_{i}=\sum_{ij} \beta c_{ij}\left[ \left( M_{i}+S_{i} \right)+k(A_{i}+P_{i}) \right]/N_{i}$$

where $\beta$ is the infection rate per potentially infectious contact; $c_{ij}$ is the daily rate of contact between age group i and j; and $N_{i}$ is the total number in age-group *i*.

| State symbol | Meaning |
| --- | --- |
| $\boldsymbol{U}_{\boldsymbol{i}}$ | Uninfected (i = 1, 2, 3 indicates for age group 0-19 y, 20-64 y and > 65 y ) |
| $\boldsymbol{E}_{\boldsymbol{i}}$ | Exposed |
| $\boldsymbol{A}_{\boldsymbol{i}}$ | Asymptomatic |
| $\boldsymbol{P}_{\boldsymbol{i}}$ | Pre-symptomatic |
| $\boldsymbol{M}_{\boldsymbol{i}}$ | Mild symptomatic |
| $\boldsymbol{S}_{\boldsymbol{i}}$ | Severe symptomatic, ultimately will need hospitalisation |
| $\boldsymbol{Q}_{\boldsymbol{i,k}}$ | Quarantined (k = 1 for mild symptomatic, k = 2 for severe symptomatic ) |
| $\boldsymbol{H}_{\boldsymbol{i}}$ | Needing hospitalisation |
| $\boldsymbol{R}_{\boldsymbol{i}}$ | Recovered and immune |

**Table S1. Definition of state variables shown in Figure S1, and in the governing equations.**

| **Parameter** | **Meaning** | **Values** | | | **Source/Notes** |
| --- | --- | --- | --- | --- | --- |
| $\beta$ | Transmission rate | 0.064 | | | Calculated using next-generation matrix as described in ref. ^1^. Value shown here is to yield R0 = 2.5 |
| $\eta$ | Incubation rate | 0.2 /day | | | Corresponds to an average incubation period of 5 days ^2^ |
| $p^{(sym)}$ | Proportion developing symptoms | 2/3 | | | Assumption ^3^, but see table 1 in main text for ranges used in uncertainty analysis |
| $k$ | Relative infectiousness of asymptomatic vs symptomatic infection | 2/3 | | |  |
| $q$ | Rate of quarantine | Specified as part of test-led intervention scenarios | | | $1/q$ is the average delay (days) between symptom onset and isolation |
| $r$ | Rate of developing symptoms | 1.0 /day | | | Assumption, corresponds to mean pre-symptomatic duration of 1 day |
| $\gamma$ | Recovery rate for non-severe cases | 0.2 | | | Assumption, corresponds to mean infectious period of 5 days |
| $h$ | Hospitalisation rate for severe cases | 0.2 /day | | |  |
| $g$ | Susceptibility to reinfection, relative to pre-infection susceptibility | 0 | | | Assumption (that immunity is perfectly protective against reinfection), but see table 1 in main text for ranges used in the analysis |
| $\omega$ | Per-capita rate at which post-infection wanes | 1/365 | | | Assumption (corresponding to mean immunity duration of 1 year), but see table 1 in main text for ranges used in the analysis |
|  | Age groups | 0-19 y | 20-64 y | >65 y |  |
| $p_{i}^{(sev)}$ | Of symptomatic cases, proportion severe (i.e. that will need hospitalization) (i = 1, 2, 3 indicates age group) | 0.002 | 0.05 | 0.225 | Taken from estimates for severity of COVID-19 ^4^ |
| $\gamma_{i}^{h}$ | Recovery rate for severe cases (i = 1, 2, 3 indicates age group) | 0.096 | 0.091 | 0.062 | Hazard rates of both $\gamma_{i}^{h}$ and $\mu_{i}$ are calculated to yield published case fatality rates ^4^, using: $CFR_{i}=\mu_{i}/(\mu_{i}+\gamma_{i}^{h}),$ and assuming on average a 10 day stay in hospital before recovery or death |
| $\mu_{i}$ | Mortality rate for severe cases (i = 1, 2, 3 indicates for different age group) | 0.0045 | 0.0088 | 0.0385 |  |
| $N_{i}$ | Population (Urban Delhi) | 7554531 | 11954901 | 818671 | Extrapolated from the Census of India 2011 ^5^ |
| $c_{ij}$ | Connectivity matrix between age group i with age group j | \| 5.59 \| 2.57 \| 0.08 \| \| --- \| --- \| --- \| \| 2.18 \| 5.56 \| 0.08 \| \| 0.06 \| 0.12 \| 0.01 \| | | | Drawn from ref. ^6^ |
|  | Health system |  | | |  |
| -- | Hospital bed capacity | 2.8 beds per 1,000 population | | | Drawn from ref. ^7^ in the example of Delhi, taking account of bed capacity in both public and private sectors |

**Table S2: Parameters used in illustrative results in Figs 1-2**. Note that for Figs.3 – 4, giving the main results of this study, we use the uncertainty intervals listed in Table 1 in the main text. Symbols are as used in the model equations, listed in the text above.

**Figure S2. Illustration of the importance of lockdown timing,** in the impact of the lockdown. Shown are different scenarios with the same effectiveness in reducing transmission (50%), but different times-of-initiation. Overall, figures illustrate the importance of lockdown timing, independently of effectiveness, for overall impact.

**Appendix 2: Cost of Testing**

We adopted an activity-based costing approach to determine the cost of conducting a RT-PCR test. We developed a cost structure for calculating the cost of both sample collection and sample analysis. Figure S3 provides a pictorial representation of the costing approach. We determined the activities involved for sample collection and sample analysis and resources required to conduct each of these activities.


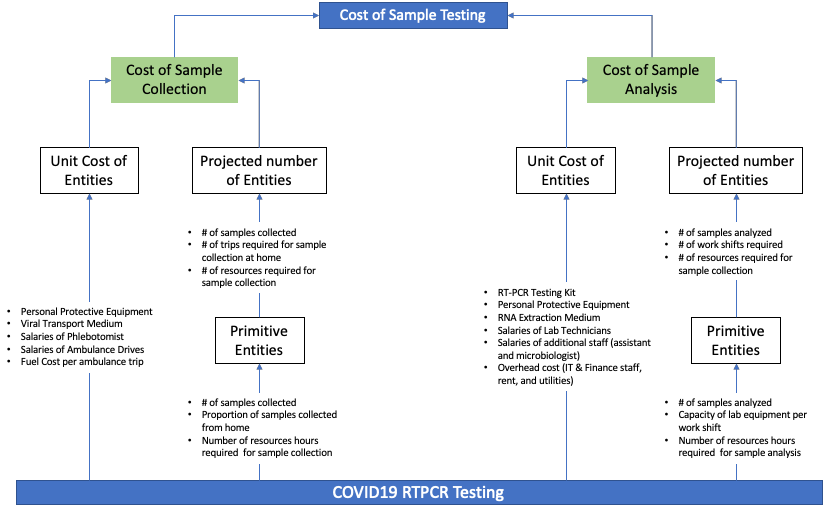


**Figure S3:** **Schematic representation of the cost structure**, with the entities and unit costs considered for sample testing.

We determined the resource requirements for each activity based on discussions with public health professionals, kit manufacturers and laboratory staff. For example, the number of ambulance staff required depends on the number of samples collected from patient homes as well as on the maximum number of samples that can be collected by an ambulance team. On the other hand, the number of test kits required depends only on the number of samples analysed. Utilities cost and management staff were assumed fixed for a laboratory irrespective of the number of tests conducted by a laboratory.

The required resources and activities scaled depending on operational parameters, and thus affect the unit cost of tests. Some of the operational parameters were:

- Throughput of each staff resource: The number of activities per unit that can be conducted by each staff will determine the number of staff resources required.
- Proportion of tests in the private sector: The staff cost for public and private sector is different and thus the unit cost of testing in public and private sector is different
- The testing capacity of a laboratory: The test analysis capacity in a single run of the machine determines the number of laboratories required to meet testing demand
- Modality of sample collection (home vs. facility): Ambulance staff is required only for samples collected from home, and the number of samples in a day collected by phlebotomist is different depending on if the samples are collected at home or in the facility.

In table S3, we list the cost components pertaining to each resource, the driving activities, and nature of each cost component (variable or fixed) with respect to increasing the number of tests. The cost structure provides unit costs for the private sector and public sector labs separately; we then derive the unit cost of testing based on the proportion of tests conducted in public and private sector.

| **Sample Collection** | | |
| --- | --- | --- |
| **Cost Category** | **Nature** | **Driving Activity** |
| **Field Staff Costs** |  |  |
| Salary of Plebotomist Staff | V | 1. Number of samples collected 2. Proportion of samples collected from patient home 3. Number of samples collected per phlebotomist in a day |
| Salary of Ambulance Staff | V | 1. Number of samples collected from patient home 2. Number of samples collected per ambulance in a day |
| **Consumables and Equipment** |  |  |
| Personal Protective Equipment | V | 1. Number of phlebotomists 2. Frequency of change of PPE |
| Viral Transport Medium | V | Number of samples collected |
| Consumables (swab, gloves, etc) | V | Number of samples collected |
| **Other Costs** |  |  |
| Fuel Cost for Ambulance | V | Number of samples collected from patient home |
| Overhead Costs (IT, Utilities, Vehicle Depreciation, etc) | F |  |
|  | | |
| **Sample Analysis** | | |
| **Cost Category** | **Nature** | **Driving Activity** |
| **Laboratory Staff Costs** |  |  |
| Salary of Lab Technician Staff | V | 1. Number of samples analysed 2. Capacity of laboratory per work shift 3. Number of work shifts |
| Salary of Lab Assistant Staff | V | Number of work shifts |
| Salary of Microbiologist Staff | V | Number of work shifts |
| **Consumables and Equipment** |  |  |
| RT-PCR Test Kits | V | Number of samples analysed |
| RNA Extraction Procedure | V | Number of samples analysed |
| Personal Protective Equipment | V | Number of lab technicians |
| Consumables (swab, gloves, etc) | V | Number of samples analysed |
| **Other Costs** |  |  |
| Overhead Costs (IT, Utilities, Vehicle Depreciation, etc) | F |  |

**Table S3: Cost components and the activities driving each of the components**

The unit cost of each resource was determined based on discussions with laboratories conducting RT-PCR tests, and the unit costs are provided in Table S4. We determined a lower bound and upper bound for each resource cost along with the most likely value, to account for the heterogenous operating models and cost structures across various laboratories and districts within the state. This resulted in a range for total cost of testing.

|  | # | **Cost Header** | **Unit of Cost** | **Lover Bound Cost/unit** | **Avg Cost/unit** | **Upper Bound Cost/unit** |
| --- | --- | --- | --- | --- | --- | --- |
| **Sample Collection** | 1 | Personal Protective Equipment | per item | ₹500 | ₹600 | ₹750 |
|  | 2 | Viral Transport Medium | per item | ₹150 | ₹200 | ₹300 |
|  | 3 | Additional Material for sample collection | per item | ₹50 | ₹50 | ₹50 |
|  | 4 | Personnel - Phlebotomist (Public Sector) | per day | ₹500 | ₹600 | ₹700 |
|  | 5 | Personnel - Phlebotomist (Private Sector) | per day | ₹600 | ₹750 | ₹900 |
|  | 6 | Personnel - Ambulance Driver | per day | ₹400 | ₹500 | ₹600 |
|  | 7 | Fuel (for Ambulance) | per km | ₹8 | ₹10 | ₹12 |
|  | 8 | Average Distance travelled for collection of sample | per sample | 10 | 15 | 25 |
| **Sample Analysis** | 1 | RNA Extraction Process | per item | ₹200 | ₹200 | ₹200 |
|  | 2 | Tesk Kit | per item | ₹1,000 | ₹1,250 | ₹1,500 |
|  | 3 | Personal Protective Equipment | per item | ₹500 | ₹600 | ₹750 |
|  | 4 | Additional Material for sample testing | per item | ₹50 | ₹50 | ₹50 |
|  | 5 | Personnel - Lab Technician (Public Sector) | per shift | ₹650 | ₹750 | ₹1,200 |
|  | 6 | Personnel - Lab Technician (Private Sector) | per shift | ₹850 | ₹1,000 | ₹1,350 |
|  | 7 | Number of lab technicians per shift |  | 2 | 2 | 3 |
|  | 8 | Number of PPEs used by lab personnel per shift |  | 2 | 3 | 4 |
|  | 9 | Microbiologist(MBBS) - Public Sector | per shift | ₹2,500 | ₹3,000 | ₹4,000 |
|  | 10 | Microbiologist(MBBS) - Private Sector | per shift | ₹3,500 | ₹4,000 | ₹5,000 |
|  | 11 | Lab Fixed Costs (IT, Rent, Electricity, Managerial Staff …) | per day | ₹7,500 | ₹10,000 | ₹15,000 |

**Table S4: Unit cost of each resource considered for calculating test cost.**

For the number of tests to be performed per day, we took model-based estimates for the required frequency of testing, in order to identify symptomatic cases of COVID-19 within 4 days of symptom onset. To allow for the existing prevalence of respiratory symptomatics with non-COVID-19 aetiologies (who would also be eligible for testing), we used estimates from the Global Burden of Disease 2017 data for the prevalence of lower and upper respiratory infections ^8^. Overall, these estimates suggested a testing effort of 111,700 samples per day in Delhi. As described in the main text, this is an artificially resource-intensive scenario, aimed at providing an upper bound on the cost of a testing programme. As a benchmark, the number of samples being tested in the whole country were around 80,000 per day as of May 15^th^. In practice, strategies for timely detection should be substantially more efficient (i.e. at lower cost) than the scenario assumed here.

Assuming that 67% of the tests occur in public sector, and 30% of the samples are collected from patient homes, we calculated the cost per test to be ₹2,388 to ₹3,770. In table S5, we provide the costs for sample collecton and analysis, along the total testing cost per day. A currency rate of $1 = ₹70 was used to find costs in US $.

|  | Lower Bound | Average | Upper Bound |
| --- | --- | --- | --- |
| Cost per sample collected | ₹477 | ₹635 | ₹859 |
| Cost per sample analysed | ₹1,911 | ₹2,320 | ₹2,911 |
| Cost per sample tested | ₹2,388 | ₹2,955 | ₹3,770 |
| Cost per sample tested (in US $) | $34.1 | $42.2 | $53.8 |
| **Total cost of Testing (in US$ million)** | **$3.81** | **$4.72** | **$6.02** |

**Table S5: The cost of testing determined by the costing approach**

**Appendix 3: Economic Loss due to Lockdown**

We selected Delhi as an illustrative example of a megacity, for the purpose of estimating the economic impact per week of lockdown. The estimated annual GDP generated by the state of Delhi is $123 billion in 2019-20 ^9,10^. The sectors forming the GDP were identified from the Economic Survey of India ^11^, and values for Delhi’s composition were based on data from from 2015-16.^12^ We used information from the lockdown guidelines issued by Government of India ^13^, media reports ^10,14–17^ and discussion with subject matter experts to classify the productivity loss into one of four categories of impact: low impact (10% loss in productivity), medium impact (50% loss in productivity), high impact (75% loss in productivity), and closed (complete shutdown of activities with 100% loss in productivity). We created three scenarios (optimistic, realistic and pessimistic), with productivity loss classified differently in each scenario. In table S6, the classification of each sector is illustrated.

| **Level** | **Sector** | **% of GDP** | **Optimistic** | **Realistic** | **Pessimistic** |
| --- | --- | --- | --- | --- | --- |
| 1 | Agriculture Sector | 1.00 |  |  |  |
| 1.1 | Agriculture, forestry & fishing | 1.00% |  |  |  |
| 1.11 | Crops | 0.62% | Low | Medium | Medium |
| 1.12 | Livestock | 0.26% | High | Closed | Closed |
| 1.13 | Forestry & logging | 0.07% | High | Closed | Closed |
| 1.14 | Fishing and aquaculture | 0.05% | High | Closed | Closed |
| 2 | Industry Sector | 9.00% |  |  |  |
| 2.1 | Mining & quarrying | 0.00% | Closed | Closed | Closed |
| 2.2 | Manufacturing | 3.00% |  |  |  |
| 2.21 | Food Products, Beverages and Tobacco | 0.27% | High | High | High |
| 2.22 | Textiles, Apparel and Leather Products | 0.41% | High | Closed | Closed |
| 2.23 | Metal Products | 0.32% | High | Closed | Closed |
| 2.24 | Machinery and Equipment | 0.71% | High | High | Closed |
| 2.25 | Other Manufactured Goods | 1.29% | Medium | High | High |
| 2.3 | Electricity, gas, water supply & other utility services | 1.00% | Low | Low | Low |
| 2.4 | Construction | 5.00% | High | High | Closed |
| 3 | Services Sector | 90.00% |  |  |  |
| 3.1 | Trade, repair, hotels and restaurants | 17.00% |  |  |  |
| 3.11 | Trade & repair services | 15.50% | High | Closed | Closed |
| 3.12 | Hotels & restaurants | 1.50% | High | High | High |
| 3.2 | Transport, storage, communication & services related to broadcasting | 6.00% |  |  |  |
| 3.21 | Railways | 0.68% | High | Closed | Closed |
| 3.22 | Road transport | 2.79% | Medium | Medium | High |
| 3.23 | Water transport | 0.04% | Medium | High | High |
| 3.24 | Air transport | 0.13% | Closed | Closed | Closed |
| 3.25 | Services incidental to transport | 0.67% | High | High | High |
| 3.26 | Storage | 0.04% | Low | Low | Low |
| 3.27 | Communication & services related to broadcasting | 1.64% | Low | Medium | Medium |
| 3.3 | Financial, real estate & prof servs | 51.00% |  |  |  |
| 3.31 | Financial services | 13.27% | Low | Medium | Medium |
| 3.32 | Real estate, ownership of dwelling & professional services | 37.76% | High | High | Closed |
| 3.4 | Community, social & pers. Servs | 16.00% |  |  |  |
| 3.41 | Public administration & defence | 3.00% | Low | Low | Low |
| 3.42 | Other services | 13.00% | Medium | Medium | High |

**Table S6: Assessed impact to each sector of economy for the state of Delhi.**

Based on the impact categorization of each sector, we found a productivity loss of 58% in the optimistic scenario, 68.9% in the realistic scenario, and 83.7% in the pessimistic scenario. Thus, applying the three scenarios, we estimated the daily loss to GDP due to lockdown to be $196 million in the optimistic scenario, $233 million in the realistic scenario, and $283 million in the pessimistic scenario.

**Bibliography**

1. Van Den Driessche, P. & Watmough, J. Reproduction numbers and sub-threshold endemic equilibria for compartmental models of disease transmission. *Math. Biosci.* **180**, 29–48 (2002).

2. Lauer, S. A. *et al.* The incubation period of coronavirus disease 2019 (CoVID-19) from publicly reported confirmed cases: Estimation and application. *Ann. Intern. Med.* **172**, 577–582 (2020).

3. Ferguson, N. M., Laydon, D., Gemma, N.-G. & et al. *Impact of non-pharmaceutical interventions (NPIs) to reduce COVID-19 mortality and healthcare demand*. (2020). doi:https://doi.org/10.25561/77482

4. Verity, R. *et al.* Estimates of the severity of coronavirus disease 2019: a model-based analysis. *Lancet Infect. Dis.* **20**, 669-677 (2020). doi:10.1016/s1473-3099(20)30243-7

5. Census of India. *Census of India 2011 META DATA*. *Office of the Registrar General & Census Commissioner, India* (2011). doi:10.2105/AJPH.2010.193276

6. Prem, K., Cook, A. R. & Jit, M. Projecting social contact matrices in 152 countries using contact surveys and demographic data. *PLoS Comput. Biol.* **13**(9):e1005697 (2017). doi:10.1371/journal.pcbi.1005697

7. Delhi Planning Department. *Economic Survey of Delhi 2018-19*. (2020).

8. Global Burden of Disease Collaborative Network. *Global Burden of Disease Study 2017 (GBD 2017) Results*. (2018).

9. International Monetary Fund. *World Economic Outlook*. (2019).

10. KPMG. *Potential impact of COVID-19 on the Indian economy*.

11. Ministry of Finance. *Economic survey of India 2019-2020*. (2020).

12. Indicus Analytics. *District GDP of India 2015-16: Premium Plus Version [Data File]*.

13. Ministry of Home Affairs. *Guidelines on the measures to be taken by Misistries/Departments of Government of India, State/Union Territory Governments and State Union Territory Authorities for containment of COVID19 Epidemic in India.* (2020).

14. Business Today. Coronavirus crisis: Lockdown to wipe off Rs 10 lakh crore from economy, warns HDFC Bank report. (2020).

15. Money Control. Nationwide lockdown to cost Indian economy $4.64 bn every day: Acuite Ratings.

16. The Economic Times. India can avoid devastation only if, carefully, economic activity is normalised and lockdown curbs removed. (2020).

17. Coronavirus: India may lose Rs 8 lakh crore of GDP to lockdown. *Deccan Herald* (2020).
